# Evaluating Fishing Ban Effectiveness Through Spatiotemporal Changes in Genetic Diversity of *Parabramis pekinensis*

**DOI:** 10.1002/ece3.72775

**Published:** 2026-04-06

**Authors:** Zhiwen Wang, Denghua Yin, Jie Liu, Pan Wang, Silei Liu, Zhongjia Huang, Wenwen Li, Mingxue Cao, Kai Liu

**Affiliations:** ^1^ Key Laboratory of Freshwater Fisheries and Germplasm Resources Utilization, Ministry of Agriculture and Rural Affairs, Freshwater Fisheries Research Center Chinese Academy of Fishery Sciences Wuxi China; ^2^ National Demonstration Center for Experimental Fisheries Science Education Shanghai Ocean University Shanghai China; ^3^ School of Marine Technology and Environment Dalian Ocean University Dalian China

**Keywords:** genetic diversity, genetic structure, *Parabramis pekinensis*, spatiotemporal changes, Yangtze River fishing ban

## Abstract

To scientifically evaluate spatiotemporal changes in genetic diversity of the bream ( *Parabramis pekinensis* ) population in the lower Yangtze River following the Yangtze fishing ban, temporal genetic diversity trends (2022–2024) were analyzed using mitochondrial *Cytochrome b* (*Cytb*) from three geographical groups (Anqing, Dangtu, Changshu), while genetic differentiation and gene flow patterns among seven geographical groups (Anqing, Tongling, Wuhu, Dangtu, Nanjing, Zhenjiang, Changshu) in 2024 were assessed through whole‐genome resequencing. The analysis exhibited a fluctuating upward trend interannually in genetic diversity: Haplotype diversity (*H*_*d*_) increased from 0.918 to 0.957, total haplotypes rose from 43 to 50, shared haplotypes expanded from 8 to 12, and nucleotide diversity (*P*_*i*_) grew from 0.00283 to 0.00295. These metrics indicate that the overall genetic diversity of the population is maintained at a moderate level (0.5 < *H*_*d*_ < 1.0, 0.001 < *P*_*i*_ < 0.005); conversely, genetic differentiation exhibited a declining trend over time, with the fixation index (*F*_*st*_) decreasing from 0.00775 to zero, reflecting substantially enhanced genetic connectivity. Among the seven geographical groups sampled in 2024, minimal genetic differentiation was observed, with pairwise *F*_*st*_ values ranging between 0 and 0.0032. And Principal Component Analysis (PCA) revealed extensive cluster overlap without geographical segregation. These results indicate negligible population structure and unrestricted gene flow among bream populations in the lower Yangtze River. It provides critical molecular genetic evidence for the comprehensive evaluation of the fishing ban's effectiveness.

## Graphical abstract

Based on spatiotemporal changes in the genetic diversity of *Parabramis pekinensis* populations in the lower Yangtze River (2022–2024), this study provides pioneering evidence that the 10‐year fishing ban has not only significantly enhanced genetic diversity but also effectively mitigated geographical barriers, thereby strengthening gene flow connectivity. These molecular metrics collectively demonstrate the ban's effectiveness in restoring fishery resources and offer critical genetic evidence for evaluating its conservation efficacy.

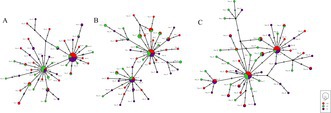

## 1 Introduction

**FIGURE 1:**
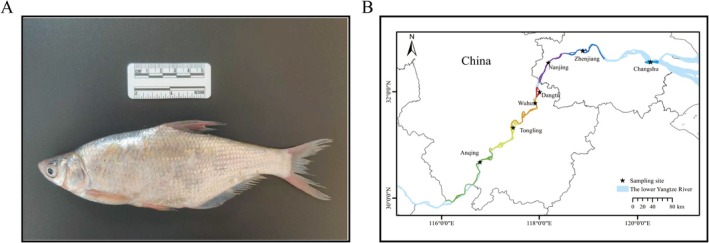
Adult *Parabramis pekinensis* and sampling sites in the lower Yangtze basin. (A) Adult bream ( *P. pekinensis* ) photographed in the Nanjing Yangtze Finless Porpoise Protected Area; (B) distribution of sampling sites of bream in the lower Yangtze River.

*Parabramis pekinensis* (Figure 1A) belongs to family Cyprinidae and is widespread throughout the Yangtze River basin, including its connected lakes and affiliated water systems (Yang et al. 2019). It is also an important freshwater economic fish in the middle‐lower Yangtze River. Bream reaches sexual maturity at 2 years and reproduces through pelagic eggs, requiring specific hydrological conditions for spawning. Its reproductive strategy utilizes hydrodynamic forces to facilitate egg drift, hatching, and dispersal. Consequently, it serves as a key bioindicator of hydrological connectivity in the Yangtze River system (Liu et al. 2017). Over the past half‐century, severe ecosystem degradation in the Yangtze Basin has been accompanied by systemic declines in bream populations. Regional biomass assessments reveal an over 80% decline in fish stocks within the Yangtze River basin (Wang et al. 2022). Specifically, populations of bream show resource depletion accompanied by juvenilization and a reduction in average body size across their distribution range (Liu et al. 2019; Dong et al. 2023). In response, China's Ministry of Agriculture and Rural Affairs implemented a decadal fishing ban (2020–2030) to facilitate ecosystem rehabilitation and critical species recovery (Mei et al. 2020; Wang et al. 2022). As such, the population dynamics of bream serve as direct metrics for evaluating ban efficacy while reflecting broader ecosystem structural–functional integrity. This provides a crucial scientific basis for maintaining the ecological balance of the Yangtze River and formulating precise conservation strategies.

Post‐ban monitoring reveals positive recovery trends in Yangtze aquatic biodiversity (Wang et al. 2023). Preliminary ecological monitoring shows that the resource abundance of bream has increased since the fishing ban, with its ecological dominance restored and the population miniaturization trend effectively halted (Shi et al. 2024). However, conventional metrics (e.g., biomass, size distributions) inadequately assess germplasm characteristics—particularly genetic diversity and population structure—which are critical for evaluating sustainable recovery (Bolnick et al. 2011). Thus, determining whether genetic diversity has increased alongside population recovery is essential for comprehensively evaluating the ban's effectiveness and the sustainability of population recovery trends.

Current research on bream is primarily focused on ecological adaptation (Luo et al. 2024), particularly within physiological ecology, including hypoxia tolerance (He et al. 2015), behavioral traits (Tang et al. 2017), nutritional requirements (Xu, He, et al. 2025), and heavy metal bioaccumulation (Jia et al. 2016). Despite significant advances in bream genomics—including the publication of its mitochondrial genome sequence (Zhang et al. 2014) and chromosome‐level reference genome (Gu et al. 2025) providing foundational resources for population genetics and phylogenetic analyses—systematic studies on germplasm characteristics and population genetics remain limited and present notable research gaps. Pre‐ban molecular investigations relied on low‐resolution methods (e.g., RAPD; Li, Tang, et al. 2008; Li, Yu, et al. 2008) or localized sampling designs (Yang et al. 2010; Chen et al. 2016; Liu et al. 2019), failing to effectively assess population dynamics after the fishing ban. Furthermore, with the increasing adoption of genomic technologies, single nucleotide polymorphisms (SNPs) have been widely applied as novel molecular markers in genetic diversity assessment and population structure analysis, owing to their advantages, including high abundance, genome‐wide distribution, and high‐throughput detection capabilities (Lind et al. 2019; Cui et al. 2024). However, research utilizing SNP markers within the bream remains relatively limited. Current studies are predominantly confined to analyses of specific captive populations (Shi et al. 2025). Consequently, comprehensive population‐level investigations encompassing broader wild populations or across different populations are notably lacking. This gap is particularly critical given that the fishing ban may reshape genetic architecture through population expansion and distribution shifts (Yang et al. 2023). Therefore, comprehensive molecular‐level analysis of genetic changes during the ban period is imperative. Such analysis will provide crucial molecular evidence for holistic evaluation of the Yangtze fishing ban's conservation efficacy.

Collectively, despite demographic recovery in this ecologically pivotal species, the short‐term genetic consequences of the fishing ban are unresolved. Current policy evaluations prioritize abundance metrics, overlooking genetic diversity as a deeper indicator of restoration efficacy. This study, therefore, employs high‐resolution spatiotemporal sampling and genomic markers to address two objectives: (1) Quantify temporal trends in genetic diversity (2022–2024) in the lower Yangtze bream population; (2) Resolve spatial genetic structure across the basin. Our findings provide critical molecular evidence for ban assessment and establish a framework for long‐term genetic monitoring.

## 2 Materials and Methods

### 2.1 Sample Collection

From April to July in 2022, 2023, and 2024, a total of 270 bream samples were collected annually from three localities in the lower Yangtze River: Anqing (AQ), Dangtu (DT), and Changshu (CS), with 30 individuals per site per year. These samples were utilized for *cytochrome b* (*Cytb*) gene analysis. Separately, to support genomic resequencing analysis, 210 samples were collected from seven localities—AQ, Tongling (TL), Wuhu (WH), DT, Nanjing (NJ), Zhenjiang (ZJ), and CS—during the period from April to November 2024, with 30 individuals collected at each site (Figure 1). Sampling employed multi‐mesh trammel nets (≥ 3 layers; 200 m length × 2 m height, mesh sizes: 2/6/10/12 cm; 50 m per mesh size) and serial fyke nets (18 m length × 0.45 m width × 0.33 m height, 0.8 cm mesh; dimensions adjusted for water depth). Dorsal muscle tissues were collected from bream and preserved in 95% ethanol at −20°C for DNA extraction.

The experimental samples were obtained through routine surveys under the Monitoring of Aquatic Resources in Key Waters of Anhui Province Project and Monitoring of Aquatic Resources in the Mainstem Yangtze River, Jiangsu Section. (Fishing License Number: Anhui Fishery Special Permit [2022] 001, [2023] 002, and [2024] 001; Jiangsu Fishery Special Permit [2022] ZT‐900002, [2023] ZT‐900001, and [2024] ZT‐000019). All sampling procedures were conducted in strict accordance with the guiding principles of the National Standard “Guidelines for the Welfare and Ethical Review of Laboratory Animals” (GB/T 35892–2018) issued by the National Technical Committee for Laboratory Animal Standardization (SAC/TC281).

### 2.2 DNA Extraction and Amplification

Total genomic DNA was extracted from 50 mg of muscle tissue using TSINGKE Tissue DNA Kit, with concentration/purity verified by microspectrophotometry.

Mitochondrial *Cytochrome b* (*Cyt b*) was amplified with universal cyprinid primers: F‐L14724: 5′‐GACTTGAAAAACCACCGTTG‐3′; R‐H15915: 5′‐CTCCGATCTCCGGATTACAAGAC‐3′. PCR reactions (20 μL) contained: 2× Hieff PCR Master Mix (10 μL), ddH_2_O (7 μL), Primers (10 μM; 1 μL each), DNA template (1 μL). Thermal cycling: 95°C/5 min (initial denaturation); 35 cycles of 94°C/30 s, 58°C/30 s, 72°C/45 s; final extension: 72°C/10 min. Amplicons were electrophoresed (1% agarose gel) and bidirectionally sequenced (Sangon Biotech Shanghai Co. Ltd.).

### 2.3 Library Construction and Data Quality Control

Genomic DNA samples meeting quality standards were randomly sheared into fragments of 300–350 bp using ultrasonication. Subsequent library preparation steps included end repair, adenylation of 3′ ends, adapter ligation, and PCR amplification to enrich target fragments. Libraries passing Qubit quantification were subjected to paired‐end sequencing on the DNBSEQ‐T7RS platform. Raw sequencing reads underwent quality control using fastp software. Adapter sequences were removed, and low‐quality reads were filtered based on the following criteria: reads with > 40% of bases below Q15, reads containing > 5 ambiguous bases (N), and shorter than 50 bp. The resulting high‐quality clean reads were used for downstream analysis.

For the resequencing analysis, the reference genome of *P. pekinensis* from the NCBI database (Accession: GCA_041684115.1), Link: https://www.ncbi.nlm.nih.gov/datasets/genome/GCA_041684115.1/. Clean reads were aligned to the reference genome using BWA. PCR duplicates were identified and removed using Picard. Initial variant calling (SNPs and InDels) was performed using GATK (Brouard and Bissonnette 2022). Variants were filtered using the following criteria: SNPs: QD < 2.0 || FS > 60.0 || MQ < 40.0 || MQRankSum < −12.5 || ReadPosRankSum < −8.0; InDels: QD < 2.0 || FS > 200.0 || ReadPosRankSum < −20.0. To ensure robustness in population‐level analyses, additional population‐based filtering was applied to the quality‐filtered variants using vcftools with the parameters: ‐‐maf 0.05 ‐‐min‐alleles 2 ‐‐max‐alleles 2 ‐‐min‐meanDP 5 ‐‐max‐missing 0.75. Finally, variants (SNPs and InDels) were annotated using ANNOVAR, and annotation statistics were generated.

### 2.4 Data Analysis

Mitochondrial *Cyt b* sequences were confirmed by BLAST search against the NCBI database. Sequence alignment and trimming were performed using MEGA 7 (Kumar et al. 2016). Genetic diversity indices were calculated using DnaSP v5.10.01 (Librado and Rozas 2009), including: number of haplotypes (*H*), haplotype diversity (*H*_*d*_), nucleotide diversity (*P*_*i*_), and the average number of nucleotide differences (*K*). Haplotype files were generated for export. Haplotype network construction was performed in PopART 1.7 (Leigh and Bryant 2015). Genetic differentiation between populations was assessed by calculating pairwise *F*_*st*_ values using Arlequin v3.5.2 (Excoffier and Lischer 2010), with significance tested using 1000 permutations.

For nuclear SNP data, linkage disequilibrium (LD) pruning was performed using Plink with the parameters ‐‐indep‐pairwise 50 10 0.1 (Purcell et al. 2007). A neighbor‐joining (NJ) phylogenetic tree was constructed from a pairwise distance matrix generated by VCF2Dis (Xu, Xu, et al. 2025) to infer population divergence and potential admixture. Principal Component Analysis (PCA) was conducted using VCF2PCACluster (He et al. 2024) to visualize population structure. To infer population genetic structure, we performed ancestry component analysis using ADMIXTURE and tested a range of possible ancestral populations (K) from 1 to 10. The model with the lowest cross‐validation (CV) error was selected as the most representative of the underlying population structure, in accordance with the software's standard recommendation (Alexander et al. 2009). Genome‐wide pairwise *F*_*st*_ values were calculated using a sliding window approach implemented in vcftools, with a window size of 200 kb and a step size of 2 kb. The biological interpretation of the *F*_*st*_ values was based on the widely accepted empirical standards in population genetics (Wright 1978), where the *F*_*st*_ coefficient ranges from 0 to 1, with 0 indicating no genetic differentiation and 1 indicating complete fixation of alleles between populations.

## 3 Results

### 3.1 Sequencing Information

For the mitochondrial Cytb dataset, a total of 270 sequences were obtained from 270 samples of *P. pekinensis* . The aligned sequence length was 992 bp, with an average base composition of 55.7% for A+T and 44.2% for G+C.

For the genomic data, all 210 samples exhibited high sequencing quality, with a mean mapping rate of 99.42%, a mean depth of 10.89×, and a mean coverage of 96.45% of the reference genome. A total of 42,348,628 raw SNPs were initially identified (Table 1). Following stringent quality filtering, 39,956,939 high‐confidence SNPs were retained. Subsequent filtering for population genetic analysis yielded a final set of 7,872,173 high‐quality polymorphic sites (Table 2).

**TABLE 1:** Summary of sequencing quality statistics for the 210 resequenced samples.

Sample number	Mean MapRate (%)	Mean depth (*X*)	Mean coverage (%)
210	99.42	10.89	96.45

**TABLE 2:** Number of single nucleotide polymorphisms (SNPs) identified and retained.

Type	Raw number	Quality filtering number	Pop filtering number
SNP	42,348,628	39,956,939	7,872,173

### 3.2 Analysis of Genetic Diversity

The calculated genetic diversity is presented in Table 3. The *Pi* of bream in the lower Yangtze River was 0.00283 in 2022, 0.00258 in 2023, and 0.00295 in 2024, indicating consistently moderate levels of nucleotide diversity across all years (0.001 < *P*_*i*_ < 0.005). While a decrease in *P*_*i*_ was observed in 2023, the overall interannual trend showed a fluctuating increase. *Hd* was high throughout the study period (*H*_*d*_ > 0.9), with values of 0.918 in 2022, 0.950 in 2023, and 0.957 in 2024, demonstrating a steady increasing trend over time.

**TABLE 3:** Genetic diversity of bream populations sampled in the lower Yangtze River during 2022**–**2024.

Populaton	Number of successfully sequenced samples	Haplotypes (*NHap*)	Haplotype diversity (*Hd*)	Nucleotide diversity (*Pi*)	Average number of nucleotide differences (*K*)
2022	90	43	0.918	0.00283	2.77
2023	90	47	0.950	0.00258	2.559
2024	90	50	0.957	0.00295	3.082

**FIGURE 2:**
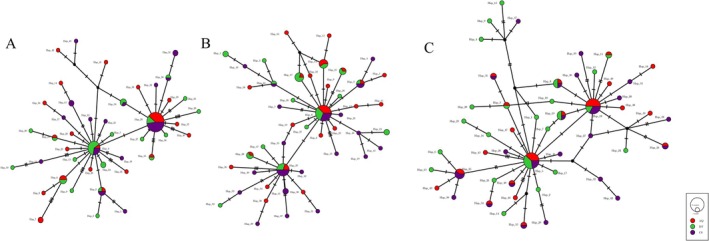
Haplotype networks of bream populations from AQ, DT and CS in the lower Yangtze River (2022–2024). (A) Haplotype networks among populations in 2022; (B) Haplotype networks among populations in 2023; (C) Haplotype networks among populations in 2024.

The haplotype network (Figure 2) revealed a shift from a predominantly star‐like distribution towards a more complex topology, indicating enhanced gene flow frequency and complexity among populations. The number of haplotypes increased from 43 in 2022 to 47 in 2023 and further to 50 in 2024. Concurrently, the number of shared haplotypes increased from 8 to 12, and the number of haplotypes shared among all populations increased from 3 to 4. Additionally, detailed variant information for each haplotype is provided in the Table S1 (2022), Table S2 (2023), and Table S3 (2024) summarize the variant site statistics for the respective years. The emergence of new haplotypes and the increase in shared haplotypes collectively indicate a rise in the genetic diversity of bream in the lower Yangtze River.

Pairwise *F*_*st*_ values among bream populations in the lower Yangtze River from 2022 to 2024 are shown in Table 4. No significant genetic differentiation was detected between the AQ and DT populations. While differentiation between the CS population and others increased from a range of 0.0057–0.0078 in 2022 to 0.021–0.024 in 2023, it then declined to zero values in 2024. These results indicate minimal genetic differentiation among bream populations in the lower Yangtze River. Moreover, concomitant with interannual population expansion and dispersal, genetic connectivity has increased among populations.

**TABLE 4:** Temporal changes in fixation index (*F*_*st*_) and *p*‐values among bream populations in the lower Yangtze River (2022–2024).

	AQ	DT	CS
2022			
Anqing (AQ)	0	0.450 ± 0.027	0.207 ± 0.041
Dangtu (DT)	−0.00089	0	0.261 ± 0028
Changshu (CS)	0.00775	0.0057	0
2023			
AQ	0	0.514 ± 0.031	0.027 ± 0.019*
DT	−0.00035	0	0.045 ± 0.020*
CS	0.02425	0.02151	0
2024			
AQ	0	0.514 ± 0.031	0.982 ± 0.010
DT	−0.00083	0	0.441 ± 0.05
CS	−0.01771	−0.00206	0

*Note:* The matrix shows significance levels (*p*‐values) above the diagonal and pairwise FST estimates below the diagonal.

* *p* ≤ 0.05.

### 3.3 Analysis of Genetic Structure

**FIGURE 3:**
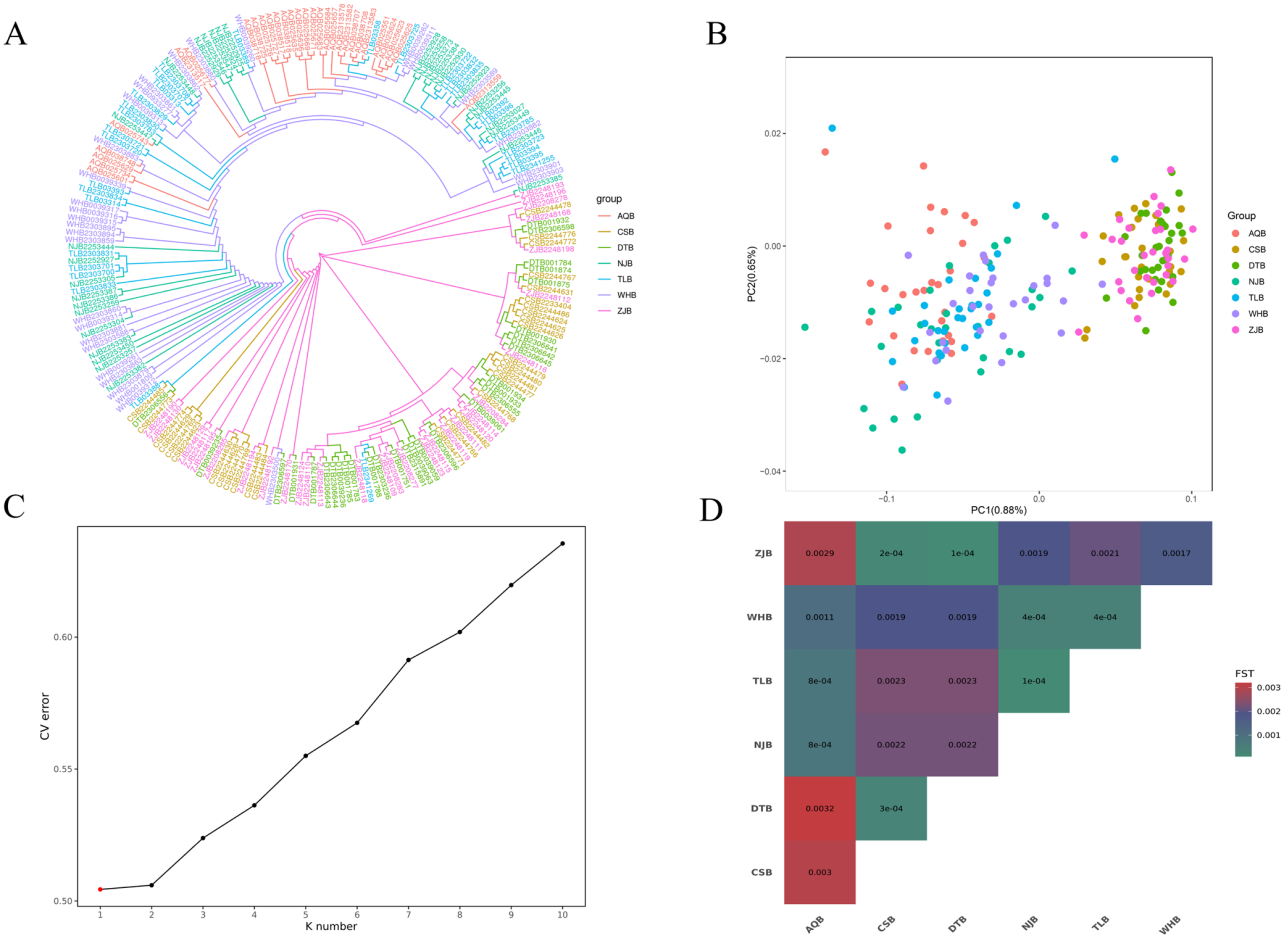
Genetic structure analysis of bream populations in the lower Yangtze River. (A) Phylogenetic tree reconstructed from resequencing data; (B) Principal component analysis (PCA) with PC1 and PC2 axes; (C) Shows the cross‐validation error plot, with the *K*‐value on the *x*‐axis and the corresponding error value on the *y*‐axis; (D) Pairwise *F*_*st*_ values among seven geographic subpopulations.

Phylogenetic analysis based on resequencing data revealed that the entire bream population in the lower Yangtze River descended from two ancestral haplotypes. Individuals from the same geographical location did not cluster exclusively on specific branches; instead, individuals from different geographical groups were intermingled across the two major ancestral haplotype branches (Figure 3A). This pattern indicates a lack of significant genetic differentiation among the seven sampled geographical groups. Principal Component Analysis (PCA) showed a very low cumulative variance contribution rate for the principal components (≤ 0.88%). The genetic structure among the seven geographical groups exhibited substantial overlap in the PCA plot, with no discernible clustering based on geography or genetics (Figure 3B). Ancestry component analysis, based on minimizing the cross‐validation (CV) error, identified *K* = 1 as the optimal number of ancestral clusters. At *K* = 1, all individuals exhibited highly homogeneous genetic composition (Figure 3C), indicating that all seven geographical groups share a single ancestral genetic background and have not formed differentiated subpopulations. Furthermore, pairwise *F*_*st*_ values among the seven geographical groups ranged from 0 to 0.0032 (Figure 3D). These findings confirm the absence of significant genetic differentiation among the lower Yangtze River bream populations and are indicative of high levels of ongoing gene flow.

## 4 Discussion

### 4.1 Post‐Ban Trends in Genetic Diversity

Since the implementation of the Yangtze River fishing ban, species richness, including that of economically important fish like bream, has generally recovered, accompanied by population expansion (Wang et al. 2023; Du et al. 2025). Studies have also indicated that the dominant status of bream, which was previously replaced by small‐bodied fish pre‐ban, has been effectively restored post‐ban (Dong et al. 2023; Shi et al. 2024). This suggests a positive impact of the fishing ban on enhancing bream genetic diversity, reflecting an increasing population base post‐ban. Moreover, these trends align closely with concurrent surveys of juvenile fish resource abundance (based on our unpublished internal data), which show an increasing trend. Abundance monitoring revealed a significant 63.3‐fold increase from the 2019 (2.13 ind/m^3^) baseline to 2024, with a fluctuating upward trajectory observed between years. Fluctuations in resource abundance directly reflect changes in population size (Li et al. 2025); the interannual increase in lower Yangtze bream abundance provides strong ecological corroboration for the positive role of the fishing ban in protecting the species' genetic diversity.

To further evaluate the genetic status of the Yangtze lower reach bream population, we compared it to other representative pelagophil fishes in the Yangtze basin. The results indicate that the nucleotide diversity of the lower Yangtze bream was lower than that reported for *Megalobrama amblycephala* population (*P*_*i*_ = 0.00483; Dong et al. 2024) in the middle‐upper Yangtze and *Hypophthalmichthys molitrix* population (*P*_*i*_ = 0.012; Xia et al. 2022), but higher than the wild *Ctenopharyngodon idellus* population (*P*_*i*_ = 0.00143; Ouyang et al. 2021) in the same region. Collectively, these comparisons indicate that the current genetic diversity of the bream population in the lower Yangtze River is at a moderate level, reflecting a moderate population size and suggesting a correspondingly moderate potential for adaptive evolution.

Furthermore, the bream population in the lower Yangtze River exhibits a distinct genetic signature characterized by low nucleotide diversity (*P*_*i*_ < 0.005) and high haplotype diversity (*H*_*d*_ > 0.5), which is a classic signature of a historically rapid population expansion (Grant and Bowen 1998). This pattern is further supported by the unimodal shape of the nucleotide mismatch distribution (Figure S1). During such expansions, the effective population size (*N*_*e*_) increases rapidly. However, if the expansion is relatively recent, insufficient time has elapsed for new nucleotide mutations to accumulate substantially, resulting in low *P*_*i*_. Conversely, high *H*_*d*_ reflects the retention of multiple ancestral haplotypes present at the onset of the expansion throughout the subsequent population growth (Gwak and Roy 2023).

Following the fishing ban, both haplotype diversity and shared haplotypes among the bream population in the lower Yangtze River showed an increasing interannual trend. This pattern is explained by both increased detectability of rare haplotypes following population recovery and by the post‐ban enhancement of riverine connectivity, which mediated the spread of formerly private haplotypes. This finding is in agreement with the results from our analysis of genetic differentiation (*F*_*st*_): the fluctuating decline in pairwise *F*_*st*_ values indicates the development of a more robust gene flow network across the lower Yangtze basin post‐ban. We suggest that this outcome is achieved through several pathways: (1) Population expansion induced by the fishing ban reduces the effective geographical distance between groups; (2) Population expansion enhances the probability of individual migration events; (3) A larger population size helps maintain a higher reservoir of standing genetic variation (Bernos et al. 2023).

However, the critical influence of habitat factors, such as extreme hydrological conditions, on ecological recovery must be acknowledged. We observed interannual fluctuations in nucleotide diversity, with a notable decrease in *P*_*i*_ occurring in 2023. This decline coincided temporally with extremely low water levels in the Yangtze basin in 2022 (Lyu et al. 2023) followed by persistently low and dry conditions in 2023 (Chen et al. 2024; Wu et al. 2023). The 2022 extreme low water event likely caused habitat desiccation, leading to individual mortality and population reduction. Furthermore, the sustained low water levels in 2023 hindered the long‐distance migration of bream to spawning grounds, negatively impacted reproduction, and impeded the dispersal and development of pelagic eggs downstream (Li et al. 2022). Consequently, hydrological disturbance is likely the key environmental factor responsible for the sharp decline in *P*_*i*_ observed in 2023. These results suggest that sustained low water levels negatively impact bream reproduction and expansion, potentially reducing nucleotide diversity, while high‐water years are more conducive to the accumulation of genetic variation. This highlights the sensitivity of bream reproduction to hydrological conditions, a typical characteristic of pelagophil fishes (Moore and Brewer 2025). This sensitivity can lead to periodic population bottlenecks, resulting in transient reductions in *N*_*e*_ and increasing the risk of genetic drift. It suggests that stable and favorable hydrological conditions are essential for consolidating the recovery benefits of the fishing ban and achieving long‐term genetic diversity accumulation (Faulks et al. 2010).

In conclusion, as a widely distributed species, bream population dynamics and genetic diversity changes are not solely linked to population recovery driven by the ban; stable and favorable hydrological conditions are equally vital. While the fishing ban promotes population growth, facilitates increased gene exchange among the population, favors the rise of shared haplotypes, weakens inter‐population differentiation, and enables diversity accumulation. Our study also demonstrates that hydrological disturbances can rapidly erode accumulated genetic variation by inducing population bottlenecks. Consequently, evaluating the fishing ban's effectiveness requires considering the dual influences of effective population expansion and hydrological environmental fluctuations on genetic diversity.

### 4.2 Post‐Ban Spatial Distribution of Genetic Structure

Analysis of whole‐genome resequencing data revealed minimal genetic differentiation among the seven geographical groups, with pairwise *F*_*st*_ values ranging from 0 to 0.0032 (below the 0.05 threshold). This finding was consistently supported by multiple lines of evidence: the absence of distinct geographical clustering in the phylogenetic tree, the highly overlapping distribution of groups in PCA, and uniform ancestral components at *K* = 1 in population structure analysis. Collectively, these results indicate the absence of significant genetic differentiation among groups and no detectable spatial genetic structure within the lower Yangtze bream population. This finding aligns closely with pre‐ban conclusions (Chen et al. 2016), suggesting that frequent gene exchange has been persistently maintained among these populations. This pattern is characteristic of widely distributed fish species (Zhao et al. 2011) and is intrinsically linked to the biological traits and ecological strategies of bream: (1) Adult bream exhibits strong migratory ability, moving between the Yangtze mainstem, tributaries, and connected lakes. Furthermore, as typical pelagophil fishes, bream reproduction relies on flowing water for egg incubation. Crucially, spawning is not restricted to specific concentrated sites (Lončar et al. 2024) but can occur in river sections with suitable hydrological conditions, such as sufficient flow velocity and distance (Li et al. 2022). This effectively overcomes geographical barriers, facilitating gene exchange and mixed reproduction among groups from different river reaches (Ferrari et al. 2023). (2) The broad and relatively continuous distribution of bream population in the lower Yangtze minimizes opportunities for physical isolation due to geographical obstacles, providing the fundamental habitat conditions for sustaining high gene flow (Zhang et al. 2020). (3) Sampling occurred during the ten‐year fishing ban period, which likely maintained or even reinforced the historically high levels of gene flow characteristic of these populations. Therefore, the observed spatial genetic pattern represents both an intrinsic species characteristic and a positive outcome of the fishing ban in maintaining population connectivity.

In summary, the powerful dispersal capacity of bream, combined with its broad distribution, effectively overcomes potential isolation effects posed by geographical distances in the lower Yangtze, enabling frequent gene exchange that results in a genetically well‐mixed population. This spatial genetic pattern exhibits temporal continuity, and the role of the Yangtze fishing ban lies in safeguarding the population size and dispersal vitality necessary to sustain this high gene flow.

## 5 Conclusions

This study provides genetic evidence consistent with a positive population response in bream following the Yangtze River fishing ban, which is concordant with independent resource survey data indicating population recovery. Collectively, these findings offer initial, multi‐faceted insights into early‐stage demographic and genetic trends, supporting the view that the fishing ban is contributing to the ecological restoration of the Lower Yangtze River.

However, the current monitoring period (2022–2024) is relatively short and does not encompass the full duration of the ban. Sustained long‐term monitoring is essential to comprehensively evaluate conservation outcomes and provide robust scientific support for the recovery of Yangtze aquatic biodiversity. Furthermore, our results reveal that the genetic recovery process is dually regulated by historical population fluctuations and habitat conditions, particularly hydrological stressors. This underscores the need, alongside strengthening aquatic species protection, to pay increased attention to the effects of climatic and hydrological factors on ecological restoration (Zhai et al. 2019). Creating favorable hydrological conditions through scientific ecological scheduling and other habitat restoration strategies will enhance overall conservation effectiveness, which is essential for achieving systematic conservation of the Yangtze aquatic biological resources (Wang et al. 2024). In summary, this study provides dynamic scientific insights for regulating Yangtze biodiversity conservation and offers genetic‐based evidence for the comprehensive assessment of the Yangtze fishing ban's efficacy.

## Author Contributions

**Kai Liu:** conceptualization (lead), funding acquisition (lead), project administration (lead), supervision (supporting), writing – review and editing (supporting). **Zhiwen Wang:** conceptualization (supporting), data curation (equal), project administration (supporting), writing – original draft (lead). **Denghua Yin:** conceptualization (supporting), data curation (equal), project administration (supporting), writing – review and editing (equal). **Jie Liu:** investigation (equal), project administration (supporting), writing – review and editing (equal). **Pan Wang:** data curation (equal), investigation (equal). **Silei Liu:** investigation (equal), project administration (supporting), resources (equal). **Zhongjia Huang:** investigation (equal), resources (equal). **Wenwen Li:** investigation (equal), resources (equal). **Mingxue Cao:** investigation (equal), resources (equal).

## Funding

This work was supported by the Central Public‐interest Scientific Institution Basal Research Fund, CAFS (2024XT1003), Monitoring of aquatic resources in key waters of Anhui province Fund (2024BFAFZ02936) and Monitoring for Aquatic Resources in the Jiangsu Section of the Yangtze River Mainstem 2024 (JSTX‐NJ2024‐056).

## Conflicts of Interest

The authors declare no conflicts of interest.

## Supporting information


**Figure S1:** ece372775‐sup‐0001‐FigureS1.pdf.

**Table S1:** ece372775‐sup‐0002‐TableS1‐S3.docx.

## Data Availability

All the whole‐genome resequencing clean reads for *Parabramis pekinensis* were deposited in the NCBI Sequence Read Archive (SRA) under accession No. PRJNA1292993, No. PRJNA1294506, and No. PRJNA1293107.
