# Supplementary figures and images for: Evaluating Fishing Ban Effectiveness Through Spatiotemporal Changes in Genetic Diversity of Parabramis pekinensis
Source: Ecol Evol. 2026 Apr 6;16(4):e72775. doi: 10.1002/ece3.72775 (PMC13054008; doi:10.1002/ece3.72775)

A

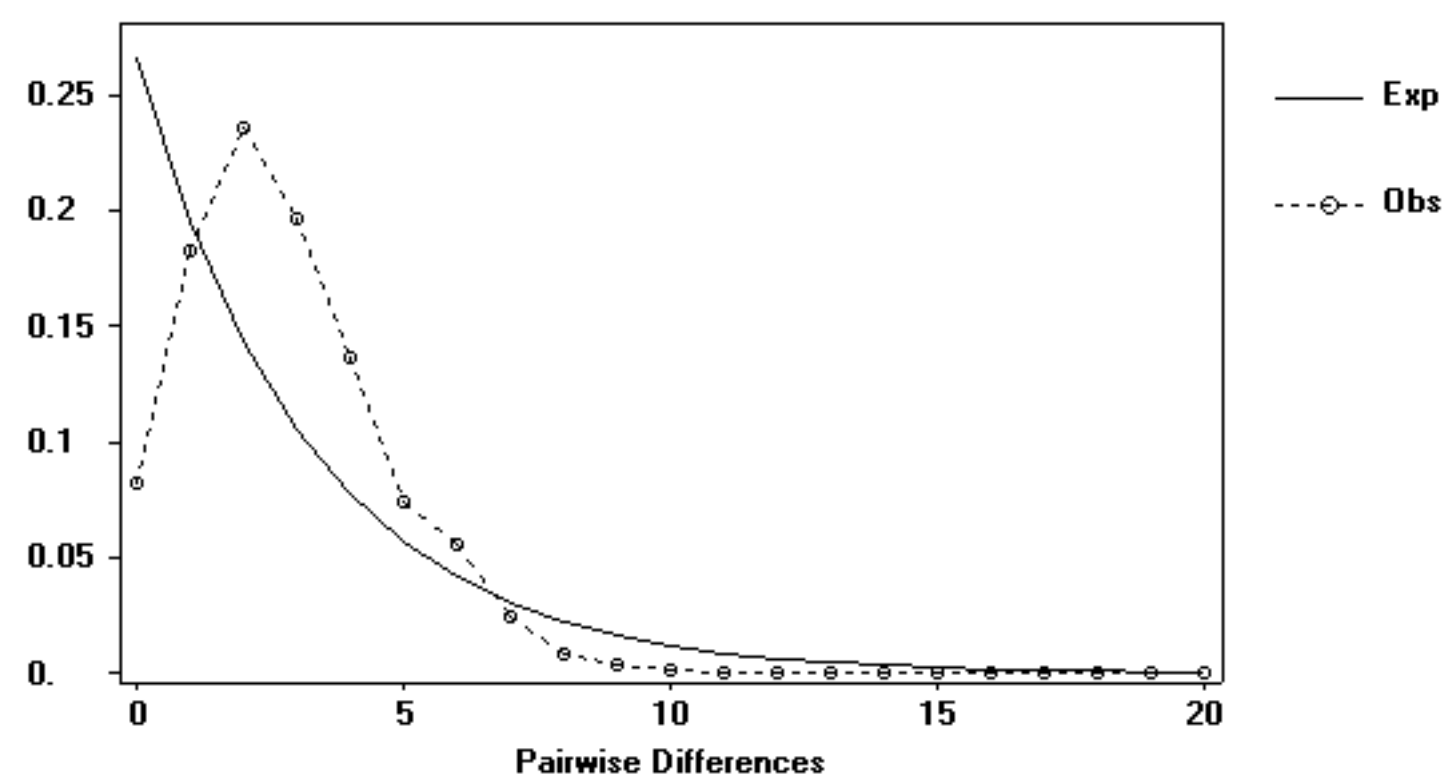

B

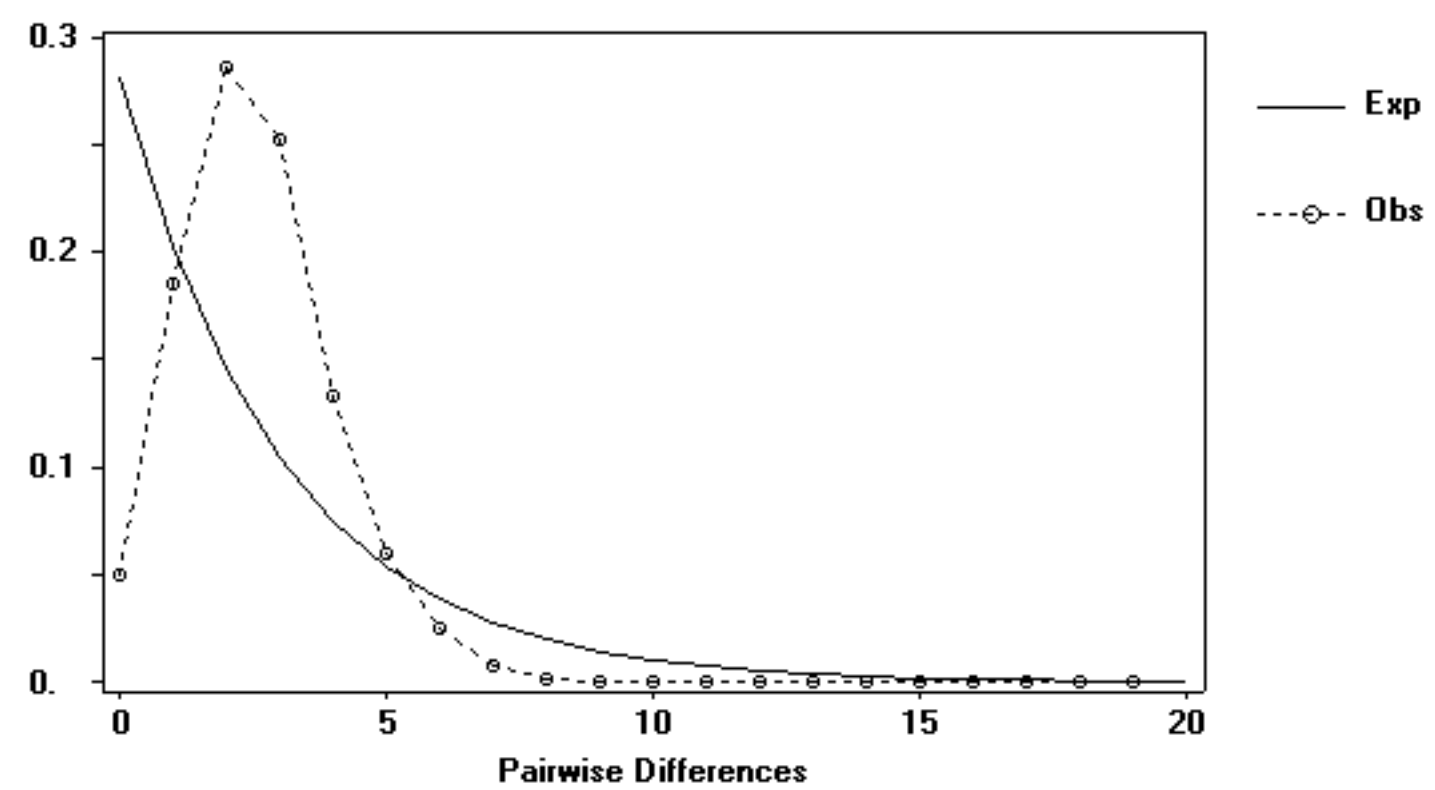

C

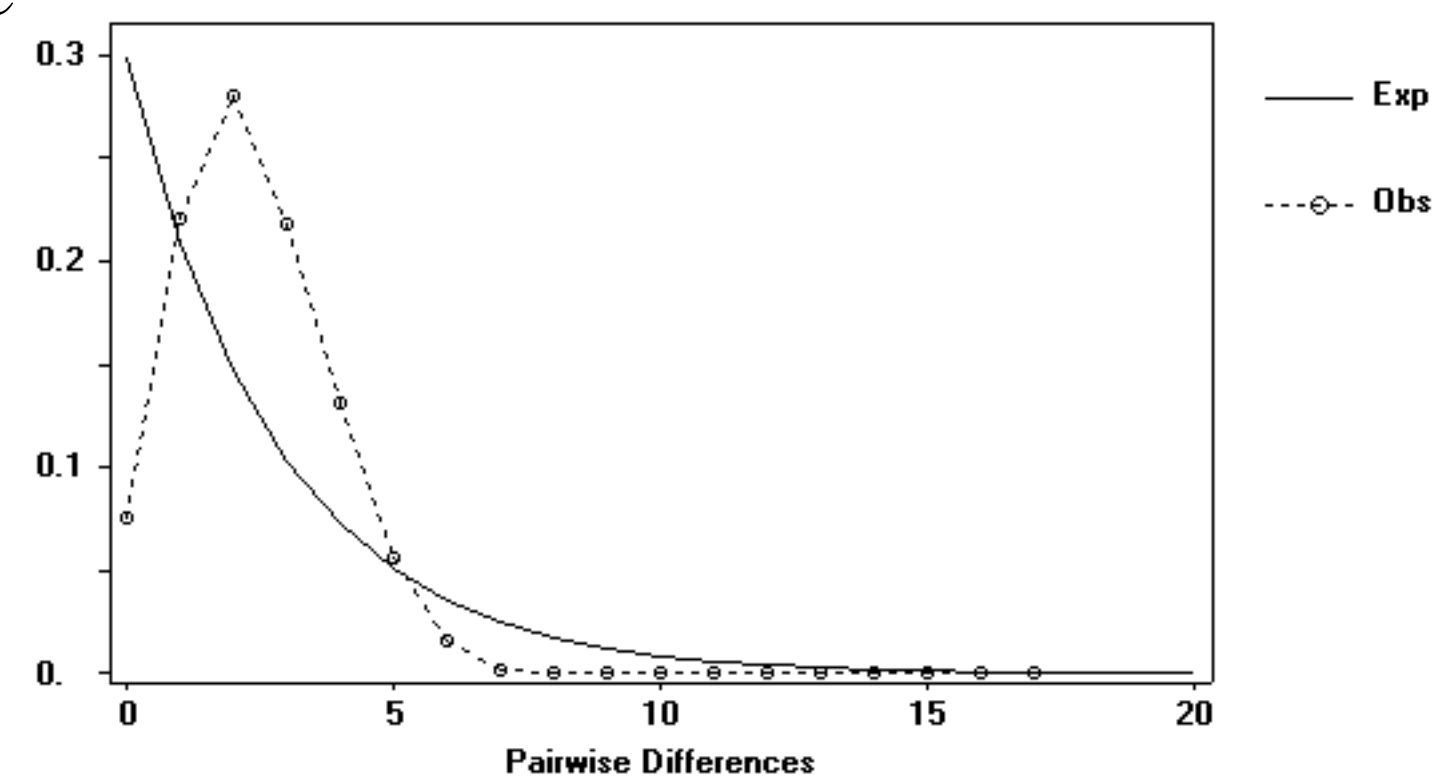

Supplement: Supplementary file 1 — Figure S1: ece372775‐sup‐0001‐FigureS1.pdf. [file ECE3-16-e72775-s002.pdf]
