# Supplementary material for: Evaluating Fishing Ban Effectiveness Through Spatiotemporal Changes in Genetic Diversity of Parabramis pekinensis
Source: Ecol Evol. 2026 Apr 6;16(4):e72775. doi: 10.1002/ece3.72775 (PMC13054008; doi:10.1002/ece3.72775)
Supplement: Supplementary file 2 — Table S1: ece372775‐sup‐0002‐TableS1‐S3.docx. [file ECE3-16-e72775-s001.docx]

**Supplementary figure 1**

Fig. S1. Analysis of the historical demographics of the *Parabramis pekinensis* population in the lower Yangtze River from 2022 to 2024.

**Supplementary tables**

We would like provide raw data results that supplement the figures in the main text.Table S1. Details of haplotype distribution in *Parabramis pekinensis* populations from Anqing (AQ), Dangtu (DT), and Changshu (CS) in the lower Yangtze River (2022)

| Hap | Sequence | AQ | DT | CS |
| --- | --- | --- | --- | --- |
| Hap_1 | CGCCACGCCCGTTATGACAAGGAAGTTCTCAATGCCTAAGCCTTCTCAATCCCTAATTCAGC | 0 | 0 | 1 |
| Hap_2 | ...................G...........................G.............. | 1 | 1 | 2 |
| Hap_3 | ...................G....................T......G.............. | 0 | 1 | 0 |
| Hap_4 | ...................G...........................G.C............ | 1 | 7 | 3 |
| Hap_5 | ...................G........................T..G.C............ | 0 | 1 | 0 |
| Hap_6 | ....G..............G...........................G.C............ | 2 | 2 | 0 |
| Hap_7 | ..T.G..............G.............A...C.A.......G.C............ | 2 | 0 | 0 |
| Hap_8 | ....G..............G..........................TG.C............ | 1 | 0 | 0 |
| Hap_9 | .....T....ACC......G...........................G.C............ | 0 | 1 | 0 |
| Hap_10 | ...................G.......T.......T.....T.C...G.C...........T | 0 | 1 | 0 |
| Hap_11 | ...................G.......T.............T.....G.C...........T | 0 | 0 | 1 |
| Hap_12 | ...................G...........................GGC............ | 0 | 0 | 1 |
| Hap_13 | ...................G....A......................G.C............ | 0 | 1 | 0 |
| Hap_14 | ...................G.............A....T........G.C.........C.. | 1 | 0 | 0 |
| Hap_15 | ...................G..................T........G.C.........C.. | 0 | 0 | 2 |
| Hap_16 | ...............A...G.A.........................G.C............ | 1 | 0 | 0 |
| Hap_17 | ...............A...G...........................G.C............ | 0 | 0 | 1 |
| Hap_18 | .................T.G...G.......................G.C............ | 0 | 0 | 1 |
| Hap_19 | ...................G...........................G.C....T....... | 1 | 1 | 0 |
| Hap_20 | ...................G......................C....G.C....T....... | 0 | 1 | 0 |
| Hap_21 | ........T..........G..G........G...............G.C............ | 1 | 0 | 0 |
| Hap_22 | .......T...........G...........................G.C............ | 1 | 0 | 0 |
| Hap_23 | ...................G...........................G.CT........... | 1 | 0 | 0 |
| Hap_24 | ...................G..............T............G.C............ | 0 | 2 | 0 |
| Hap_25 | ...................G..................G........G.C........T... | 0 | 0 | 1 |
| Hap_26 | ...T...............G...........................G.C............ | 1 | 0 | 0 |
| Hap_27 | ..............C....G...........................G.C...C........ | 0 | 1 | 0 |
| Hap_28 | ...................G...........................G.C...C........ | 9 | 3 | 11 |
| Hap_29 | ..................GG................C........C.G.C...C.G...... | 0 | 1 | 0 |
| Hap_30 | ...................G.....C.....................G.C...C........ | 0 | 1 | 1 |
| Hap_31 | ...................G.....C.....................G.C...C...C.... | 0 | 0 | 2 |
| Hap_32 | ...................G.............................C...C........ | 0 | 0 | 1 |
| Hap_33 | T..................G.........T.................G.C...C........ | 0 | 1 | 0 |
| Hap_34 | ...................G...........................G.C...C..C..... | 0 | 2 | 1 |
| Hap_35 | ................G..G........C..................G.C.T.C........ | 1 | 1 | 0 |
| Hap_36 | ...................G......C....................G.C...C........ | 0 | 1 | 0 |
| Hap_37 | ...................GA..........................G.C...C........ | 1 | 0 | 0 |
| Hap_38 | ...................G............C..............G.C...C........ | 1 | 0 | 0 |
| Hap_39 | .A.................G...........................G.C...C........ | 1 | 0 | 0 |
| Hap_40 | .........T.........G..........G................G.C...C....T... | 1 | 0 | 0 |
| Hap_41 | ...................G...........................G.C......C...A. | 1 | 0 | 0 |
| Hap_42 | ......A......G.....G...........................G.C......C..... | 1 | 0 | 0 |
| Hap_43 | ......A............G...........................G.C..T...C..... | 0 | 0 | 1 |
| Total |  | 30 | 30 | 30 |

Table S2. Details of haplotype distribution in *Parabramis pekinensis* populations from Anqing (AQ), Dangtu (DT), and Changshu (CS) in the lower Yangtze River (2023)

| Hap | Sequence | AQ | DT | CS |
| --- | --- | --- | --- | --- |
| Hap_1 | TTCCCAACCCTTCCGCTTTCATGATCAGCGTTTCACTCCTACCCTCACATTAGC | 0 | 2 | 0 |
| Hap_2 | ......G....C.....................................C.... | 0 | 1 | 0 |
| Hap_3 | ......G........................................T.C...T | 1 | 1 | 2 |
| Hap_4 | ......G....................................T...T.C...T | 0 | 0 | 1 |
| Hap_5 | ......G..................T.....................T.C...T | 1 | 0 | 0 |
| Hap_6 | ......GT.........................................C...T | 1 | 0 | 0 |
| Hap_7 | ......G.............G............................C..AT | 0 | 0 | 1 |
| Hap_8 | ......G..........................................C...T | 6 | 5 | 5 |
| Hap_9 | ......G...C......................................C...T | 1 | 0 | 0 |
| Hap_10 | ......G...........C..............................C...T | 1 | 0 | 0 |
| Hap_11 | ......G.....................T....................C.T.T | 1 | 0 | 0 |
| Hap_12 | ......G..........................................C.T.T | 1 | 0 | 0 |
| Hap_13 | .C....G..T.................A.A...................C...T | 0 | 0 | 1 |
| Hap_14 | .....GG..........................................C...T | 3 | 2 | 0 |
| Hap_15 | .....GG............................T.............C...T | 1 | 0 | 0 |
| Hap_16 | .....GG...............A................C.........C...T | 1 | 0 | 0 |
| Hap_17 | ......G...............A..........................C...T | 1 | 4 | 0 |
| Hap_18 | ......G......................................T...C.... | 1 | 0 | 0 |
| Hap_19 | ......G.T...............................T........C...T | 0 | 0 | 1 |
| Hap_20 | ......G.......................C..................C...T | 0 | 1 | 0 |
| Hap_21 | ......G.............................C............C...T | 0 | 0 | 1 |
| Hap_22 | ......G................................C.........C...T | 1 | 0 | 0 |
| Hap_23 | ......G..............C...........................C...T | 1 | 0 | 0 |
| Hap_24 | ......G.........C.......................T........C...T | 0 | 2 | 0 |
| Hap_25 | C.....G......T...................................C...T | 1 | 2 | 0 |
| Hap_26 | ......G.......A..................................C...T | 0 | 1 | 0 |
| Hap_27 | ....T.G..........................................C...T | 0 | 1 | 0 |
| Hap_28 | ......G..............................T...........CC..T | 1 | 2 | 0 |
| Hap_29 | ......G..........................................CC..T | 2 | 3 | 5 |
| Hap_30 | ......G........................C.................CC..T | 1 | 0 | 0 |
| Hap_31 | ......G...................G....C.................CC..T | 0 | 0 | 1 |
| Hap_32 | ...T..G.....T.....................G..............CC..T | 0 | 1 | 0 |
| Hap_33 | ......G.....T.....................G..............CC..T | 0 | 0 | 1 |
| Hap_34 | ......G.....................................C....CC..T | 1 | 0 | 0 |
| Hap_35 | ......G...........C..............................CC..T | 0 | 0 | 1 |
| Hap_36 | ......G..........C.T............................GCC..T | 1 | 0 | 0 |
| Hap_37 | ......G.................C.............T...T...G..CC..T | 0 | 0 | 1 |
| Hap_38 | ..T...G..........................................CC..T | 1 | 0 | 0 |
| Hap_39 | ..T...G..........................T...............CC..T | 0 | 0 | 1 |
| Hap_40 | ......G..................................T.......CC..T | 0 | 0 | 1 |
| Hap_41 | ......G.........................C................CC..T | 0 | 0 | 2 |
| Hap_42 | ......G................................A.........CC..T | 0 | 0 | 1 |
| Hap_43 | .................................................C.... | 0 | 0 | 1 |
| Hap_44 | ......G..........................................C.... | 0 | 1 | 1 |
| Hap_45 | ......G........T........................T........C...T | 0 | 0 | 1 |
| Hap_46 | ......G.................................T........C...T | 0 | 0 | 1 |
| Hap_47 | ......G................G.........................CC..T | 0 | 1 | 0 |
| Total |  | 30 | 30 | 30 |

Table S3. Details of haplotype distribution in *Parabramis pekinensis* populations from Anqing (AQ), Dangtu (DT), and Changshu (CS) in the lower Yangtze River (2024)

| Hap | Sequence | AQ | DT | CS |
| --- | --- | --- | --- | --- |
| Hap_1 | TCCAAGTCCCTCCGCTTGCGTAGACGCTCCAGCTCCTACCTATCTATGCACGCAGG | 0 | 1 | 0 |
| Hap_2 | ......................A........................A........ | 1 | 1 | 0 |
| Hap_3 | ..............................................CA........ | 1 | 0 | 1 |
| Hap_4 | ..............T......................T.........C.....G.. | 1 | 0 | 1 |
| Hap_5 | ...............................................A........ | 0 | 0 | 1 |
| Hap_6 | ......................A..................G.T...AT....... | 0 | 1 | 0 |
| Hap_7 | ....................C..........................A........ | 5 | 5 | 3 |
| Hap_8 | ......................A.....................C..A.....G.. | 0 | 0 | 1 |
| Hap_9 | ...............C.....................T.........C.....G.. | 0 | 1 | 0 |
| Hap_10 | ............................................C.CA........ | 0 | 1 | 0 |
| Hap_11 | ...........T..................G.............C..A........ | 0 | 1 | 0 |
| Hap_12 | ............................................C..A.G...... | 1 | 0 | 0 |
| Hap_13 | ...........................................T...AT....... | 0 | 1 | 0 |
| Hap_14 | ...G...........................................A......A. | 0 | 1 | 0 |
| Hap_15 | ......C...................T..........T.........C.....G.. | 0 | 0 | 1 |
| Hap_16 | ........T......................................A........ | 0 | 1 | 0 |
| Hap_17 | ..................T............................A........ | 0 | 1 | 0 |
| Hap_18 | ...............................................A........ | 0 | 1 | 0 |
| Hap_19 | ....................C.......................C..A........ | 1 | 0 | 0 |
| Hap_20 | ..........C...................................CA........ | 2 | 0 | 3 |
| Hap_21 | C..............................................A........ | 0 | 1 | 0 |
| Hap_22 | ...........................C................C..A.......A | 0 | 1 | 0 |
| Hap_23 | ........T...T..................................A........ | 0 | 0 | 1 |
| Hap_24 | ......................G...........T.........C..A.......A | 1 | 0 | 1 |
| Hap_25 | ...........................................T...AT....... | 1 | 0 | 0 |
| Hap_26 | ...................................T...........A........ | 0 | 1 | 0 |
| Hap_27 | ............................................C..A.......C | 1 | 0 | 1 |
| Hap_28 | ...............................................A........ | 0 | 1 | 0 |
| Hap_29 | ...G..................A.............C..........A........ | 1 | 0 | 0 |
| Hap_30 | ......................G....................T...AT....... | 1 | 2 | 1 |
| Hap_31 | .....A........................................CA........ | 1 | 0 | 0 |
| Hap_32 | ...............................................A...A...C | 0 | 0 | 1 |
| Hap_33 | ...................A........................C..A........ | 1 | 0 | 0 |
| Hap_34 | C..............................................A.G...... | 6 | 2 | 4 |
| Hap_35 | ...G............................T..............A........ | 1 | 0 | 1 |
| Hap_36 | .............A..............................C..A........ | 0 | 0 | 1 |
| Hap_37 | ....G................................T.........C.....G.. | 0 | 0 | 1 |
| Hap_38 | .T...........................T..............C..A.......A | 0 | 1 | 0 |
| Hap_39 | .........................A..................C..AT....... | 1 | 0 | 0 |
| Hap_40 | .................................C.............A........ | 0 | 1 | 0 |
| Hap_41 | .................A.............................A........ | 1 | 0 | 0 |
| Hap_42 | ...............................................A...A..AC | 1 | 2 | 1 |
| Hap_43 | ...............................A...........T...AT....... | 0 | 0 | 1 |
| Hap_44 | ...................A........................C..A....T... | 0 | 0 | 1 |
| Hap_45 | ..T...................................T........A........ | 0 | 0 | 1 |
| Hap_46 | ................C.......................C....T.A........ | 0 | 1 | 0 |
| Hap_47 | .......T.......................................A........ | 1 | 1 | 0 |
| Hap_48 | .......................G....................C........... | 0 | 0 | 1 |
| Hap_49 | ............................T...............C........... | 0 | 0 | 1 |
| Hap_50 | ............................................C..A..T....A | 0 | 0 | 1 |
| Total |  | 30 | 30 | 30 |
